# Compliance with Home-based Fortification Strategies for Delivery of Iron and Zinc: Its Effect on Haematological and Growth Markers among 6-24 months Old Children in North India

**Published:** 2014-06

**Authors:** Sunil Sazawal, Pratibha Dhingra, Usha Dhingra, Shilpi Gupta, Venkatesh Iyengar, Venugopal P. Menon, Archana Sarkar, Robert E. Black

**Affiliations:** ^1^Department of International Health, Johns Hopkins Bloomberg School of Public Health, Baltimore, USA; ^2^Center for Micronutrient Research, Annamalai University, Chidambaram, India; ^3^Center for Public Health Kinetics, New Delhi, India; ^4^Biomineral Sciences International, Inc., Washington, DC, USA

**Keywords:** Compliance, Fortified complementary food, Growth, Iron status, Sprinkle, Zinc

## Abstract

Compliance is a key component in successful implementation of the delivery of micronutrients among children. The present study evaluates the compliance with two home-based food fortification strategies (fortified complementary food or sprinkle) for providing iron and zinc among children aged 6-24 months. A total of 292 children were randomly allocated to receive either rice-based fortified complementary food and nutrition education (Cf=101), sprinkle and nutrition education (Mp=97), or nutrition education alone as control (Ed=94). All the enrolled children were breastfed at the beginning of the study and were advised to continue breastfeeding. Biweekly information on compliance and anthropometry was collected. Complete haemogram estimation was conducted at baseline and end of the study. Compliance with the fortified complementary food was higher compared to sprinkle (Cf=81%, Mp=64% child-days). Consumption of the fortified complementary food for 6 months resulted in a significant increase in mean haemoglobin in the intervention group compared to control group (Cf 1.29±1.6 g/dL; Ed 0.23±1.3 g/dL; p<0.001). Our results showed that fortified complementary food had higher compliance than sprinkle and is a suitable delivery mechanism for iron and zinc in preschool children.

## INTRODUCTION

Recent studies suggest that iron and zinc deficiencies coexist and are a major cause of morbidity and mortality due to infectious diseases among children in developing countries (1-3). Zinc deficiency leads to growth retardation and developmental delays in children, impedes genital development and hypogonadism (4). One of the most comprehensive reviews evaluating the effect of zinc supplementation on growth showed that zinc supplementation had a highly significant positive effect on linear growth (5). India, like other developing countries, has a high burden of micronutrient deficiencies, with almost 75% of its children suffering from iron-deficiency anaemia and over 50% of children from zinc deficiency (2,6). Evaluation of feasible and easily acceptable strategies to delivering iron and zinc is, thus, a priority.

Success of iron and zinc supplementation programmes in developing countries, like India, have been limited due to poor compliance and coverage, inadequate programme support (absence of commitment at the national and community levels), failure on the part of healthcare delivery systems, cost factors, etc. (7-10). More recently, three large trials on the effect of iron and zinc supplementation among 1-48 month(s) old children living in Africa, India, and Nepal showed that children supplemented with iron/folic acid plus zinc had higher adverse effect compared to placebo group, thus suggesting evidence of negative zinc-iron interaction for clinical outcomes and raising concerns about the safety of zinc-iron supplementation (11-13). It has been observed that, when iron and zinc are delivered through food, the interaction is less or not present and also the compliance with fortification is found better than supplementation (14). However, nutrition education programmes and strategies to promoting consumption of animal-source foods to meet the nutritional requirement of children are found difficult to implement in countries, like India (15-16).

Fortified complementary foods (17-19) and micronutrient powders (20-22) are two commonly-acceptable means of delivering micronutrients through food medium among infants and young children. Studies have shown that high compliance with fortified complementary foods has often been reflected in improved haematological and growth parameters, thereby suggesting that compliance is a key factor in determining the effectiveness of the fortification strategy for the delivery of micronutrients (21,23-24).

In the present study, we evaluated compliance with two home-based fortification strategies for delivery of iron and zinc in the form of fortified complementary food or sprinkle for a period of 6 months among 6-24 months old children. We also assessed the effects of compliance with fortification on haematological status and growth markers compared to the control group which received nutrition education alone as a secondary outcome.

## MATERIALS AND METHODS

### Study area, participants, and recruitment

The present study was conducted in Sangam Vihar, a low-income, resettlement community in North Delhi, India. The eligible children (6-24 months of age; n=325) were identified from the pre-existing surveillance database, and 292 were included for analysis if the children were between 6 and 24 months of age, consuming complementary food in addition to breastfeeding, not having any severe illnesses or severe acute malnutrition (SAM) requiring rehabilitation, and willing to stay in this study area until the next six months. Informed written consent to participate in the study was obtained from the mothers of the children who participated in the study. The study protocol was approved by the human research and ethical review committees of Annamalai University, Chidambaram, India and Johns Hopkins University, Baltimore, USA.

### Study design

#### Randomization and blinding

Cluster sampling was carried out to identify 12 clusters, wherein 25 households from each cluster were randomly selected, matched, and distributed into three study groups with 4 clusters each (Figure 1). Eligible children were selected from the clusters and randomized to receive either rice-fortified complementary food and nutrition education (Cf=101), sprinkle and nutrition education (Mp=97), or nutrition education alone as control (Ed=94). Refusals were defined as those households that refused to continue in the study because of their children not liking the fortified complementary/sprinkle food. Matching of the clusters was based on assessments of the child's characteristics. Blinding of the field staff or mothers was not possible, although people responsible for laboratory and data analyses were blinded to the group assignments.

#### Intervention

The intervention was given for six months either in the form of fortified complementary food or sprinkle delivered in sachets. Intervention was delivered monthly to the mother. The rice-based fortified complementary food was developed by Jee Vee Foods Pvt Ltd, India, using the expertise of Central Food Technologies Research Institute, Mysore, India. Each sachet contained either 20 g or 40 g of the rice-based fortified complementary food in the powder form. Children less than 1 year of age received 20-g sachet whereas children older than 1 year received 40-g sachet. Mothers were explained to reconstitute the food in water or milk to make about 100 mL or 200 mL feed to be provided once daily, in addition to the normal diet of the child. The dosage of iron and zinc in the sachets of 20 g and 40 g was 7.9 mg and 6.5 mg, and 15.9 mg and 13.0 mg respectively. Protein-energy ratio (PER) and fat-energy ratio (FER) of the fortified complementary food was 3.73 g/100 kcal and 1.87 g/100 kcal respectively.

Sprinkle was prepared by Nutriset, Malaunay, France and procured by World Health Organization (WHO) for the study. The composition of the sprinkle was similar to WHO supplement for persistent diarrhoea with additional 12.5 mg of iron and 10 mg of zinc (http://whqlibdoc.who.int/hq/1998/WHO_NUT_98.1.pdf). The value of iron was chosen on the basis of estimates that absorption of iron would be low due to the presence of dietary phytate, a potent inhibitor of iron absorption (25). Mothers in this group were advised to add a sachet of powder to the child's meal-serving (after cooking) once daily. Whereas children less than 12 months of age were provided half the quantity of the sprinkle, they were also asked to feed the home-prepared food immediately after the addition of powder as, on standing, a change in food colour was reported. Composition of fortified complementary food (in 20-g and 40-g sachet) and sprinkle powder feed are shown in Table 1.

**Figure.**
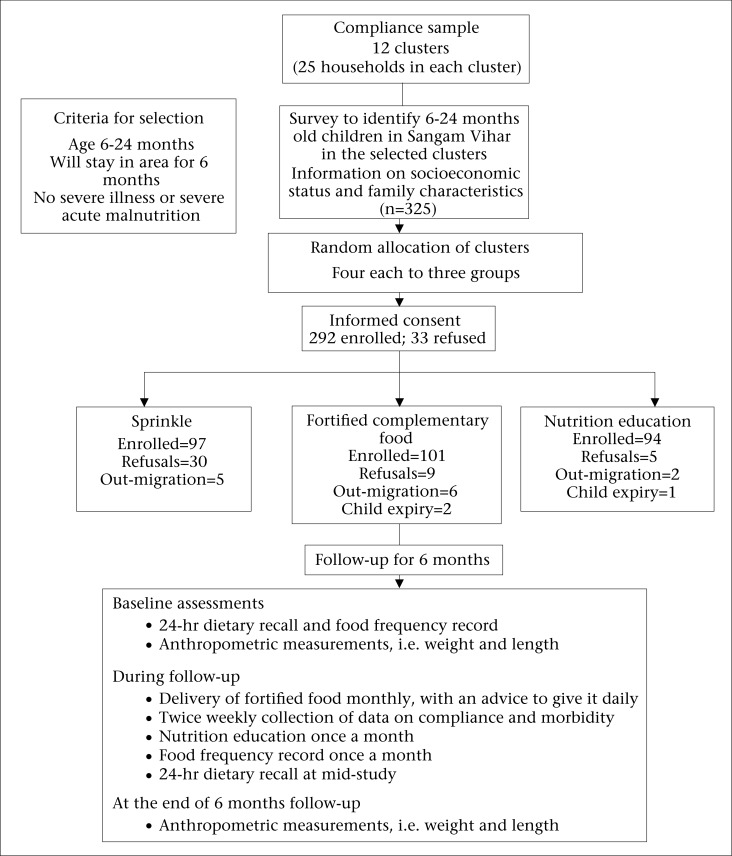
Overview of study design

Mothers were educated about the importance of micronutrients, dietary diversification methods to include foods rich in iron and zinc, and other food items rich in different micronutrients which can be easily included in the child's diet. This education was given once a month to all the three groups. In addition, all the participants were advised to continue breastfeeding.

**Table 1.** Nutrient contents of the fortified complementary food and micronutrient (sprinkle) powder

Ingredient	Fortified food: 20 g (100 mL mix)	Fortified food: 40 g (200 mL mix)	Micronutrient powder (per 1.5 g)
Energy content (kcal)	80.4	160.8	-
Protein (g)	3.0	6.0	-
Fat (g)	1.5	3.0	-
Linoleate (g)	0.18	0.36	-
Vitamin A (IU)	1,800	3,600	978.26 µg
Thiamine (mg)	0.51	1.03	0.98
Riboflavin (mg)	0.58	1.17	1.11
Niacin (mg)	6.6	13.2	13.04
Pyridoxine (mg)	0.83	1.67	1.3
Cyonocobalamine (mg)	0.21	0.42	3.91 µg
Biotin (mg)	0.11	0.22	19.57 µg
Folic acid (mg)	0.144	0.288	260.87 µg
Vitamin C (mg)	23.4	46.8	39.13
Vitamin D (IU)	145	290	6.52 µg
Vitamin E (IU)	10.5	21	9.78 µg
Calcium (mg)	138.7	277.5	494.02
Magnesium (mg)	20	40	69.13
Phosphorus (mg)	64	128	81.52
Iron (mg)	7.9	15.9	12.5
Zinc (mg)	6.5	13	10.0
Copper (mg)	1.0	2.0	1.3
Manganese (mg)	0.16	0.32	2.58
Pantothenic acid (mg)	-	-	6.52

#### Assessment

At the time of enrollment, baseline information regarding socioeconomic status, demographic profile of parents, and feeding behaviour of children, including record of dietary intake and frequency of breastfeeding, was collected. Twice weekly data on compliance with the fortification were collected through recording reported use by the caregiver, counting the number of empty sachets, mother's perception on the amount and ease with which child consumed the fortified food, whether child liked the fortified food, observation of any change in the colour, and taste or texture of the food.

At the baseline and at the end of the study, 3 mL venous blood was collected for estimating complete haemogram, using Coulter automated flow cytometer (Beckman-Coulter, Fullerton, CA, USA). The effect of intervention on iron status and anaemia was evaluated by estimating the difference in means from baseline to end of the study for each of the haematological parameters [Hb, haematocrit (HCT), mean corpuscular volume (MCV), red cell distribution width (RDW)], and the change in proportion of children with iron-deficiency anaemia.

Dietary intake assessments were performed using 24-hour dietary recall at baseline and at 3 months (mid-study). In addition, food frequency questionnaire was administered at monthly interval during the 6 months follow-up. Household measures were used for quantities, and the participating families were encouraged to serve the child's meal on standardized plates to facilitate recording. Nutritive values were calculated using standard food composition tables (26).

Weight and length were measured at baseline and at the end of the study, using electronic weighing scale (SECA, Columbia, MD), with a sensitivity of ±10 g and Shorr's infants’ length board (Shorr Productions, Onley, MD) with a sensitivity of ±0.1 cm. Anthropometric z-scores were derived using the Epi info statistical software (version 3.3.2) for various growth indicators, such as length-for-age (LAZ), weight-for-length (WLZ), and weight-for-age (WAZ), using WHO standards (27). The effect of fortification on growth parameters was evaluated by estimating the mean change in z-scores of these growth indicators (baseline to end of the study).

### Data processing and analysis

Data were analyzed using SPSS (version 18.0) (SPSS Inc., Chicago, Illinois, USA). Percentage of child-days, when the supplement was given to the child, was used in estimating the data on compliance and whether the mother and child accepted the supplements. Paired *t*-test was used in analyzing the statistical significance of mean pre-post change in the haematological markers and anthropometric measurements among the intervention groups and the control group. Anaemia was defined as Hb concentration <10 g/dL (28). Proportion of children with anaemia (Hb<10 g/dL) was calculated at baseline and at the end of study. A lower cutoff was selected instead of the WHO cutoff of 11 g/dL because majority of the iron-deficient children had Hb below 10 g/dL. Intent to treat analysis was performed, and all children enrolled were included in the analysis, irrespective of compliance.

## RESULTS

A total of 292 children, aged 6-24 months, from 12 clusters were enrolled for 6 months (Figure). Of the enrolled children, 16 were withdrawn from the study due to out-migration or death. Baseline characteristics of the three groups were similar (Table 2).

Results relating to compliance with fortification are presented in Table 3. The compliance with sustained use was 63.9% and 81.4% child-days in the sprinkle and the fortified complementary food-group respectively. Children in both the intervention groups consumed almost full sachet of the fortified food or powder in more than 80% of the child-days. Throughout the study, mothers generally reported good appetite of children (Mp: 87.7%, Cf: 92.3%).

The proportion of infants who refused fortification was greater in the sprinkle group (n=30) than the rice-based fortified complementary food-group (n=9) (Figure). Only 11% of the children in the fortified complementary food-group refused for not liking the fortified food compared to 23.7% in the sprinkle group. Usually, the sprinkle was added to milk, although it was acceptable with all other commonly-consumed complementary foods. No adverse effects relating to the interventions were reported during the study.

In the study, 24-hour dietary recall was estimated at baseline and at three months. In addition, food frequency questionnaire was administered at monthly interval. In the analysis, both comparisons: pre-post within the groups and post-comparisons between the groups were evaluated. As these comparisons were similar, we are presenting results of the end-study dietary assessments in this paper. Results showed that both sprinkle and fortified complementary food did not displace the regular dietary intake of the child as assessed by 24-hour dietary recall at mid-study and food frequency estimations after 6 months of intervention [Energy intake: (Mp 512±280; Cf 492±280; Ed 507±304 kcal)]. Effect of food fortification on the change in the haematological markers and growth indicators in two intervention groups compared to control group are shown in Table 4. Rice-based fortified complementary food resulted in significant improvement in mean haemoglobin compared to the control group. A similar pattern was observed for HCT and MCV, except for RDW which showed statistically non-significant difference. Sprinkle fortification did improve the iron status but these changes were not statistically significant. There was no significant effect of either fortification on length/weight velocity of children. Fortified complementary food-group had 67% reduction in proportion of children with anaemia (Hb<10 g/dL) compared to 27% reduction in the sprinkle group and 22% reduction in the control group.

**Table 2.** Child's characteristics, socioeconomic status, and feeding behaviour at baseline

Variable	Sprinkle (n=97)	Fortified complementary food (n=101)	Education (n=94)
Age in months (mean±SD)	16.2±5.2	16.3±5.2	15.2±5.4
Gender (M) (n, %)	57 (58.8)	51 (50.5)	50 (53.2)
Non-literate mother (n, %)	49 (50.5)	54 (53.5)	48 (51.5)
Occupation of father			
Daily wager (n, %)	28 (28.9)	25 (24.8)	21 (22.3)
Occupation of mother			
Housewives (n, %)	89 (91.8)	96 (95.0)	85 (90.4)
SES score (mean±SD)	7.21±2.68	7.05±2.58	7.62±2.64
Household type			
Nuclear (n, %)	70 (72.2)	82 (81.2)	63 (68.1)
Feeding patterns			
Milk with solids (%)	86.6	90.1	87.2
Solids (%)	13.4	7.9	12.8
Mother's perception of child's appetite			
Good (%)	66.0	68.3	70.2
Efforts required to feed			
Less than usual/None (%)	64.0	64.4	69.2
Usual (%)	36.1	35.6	30.9

**Table 3.** Mother's/child's experience with fortification during the visits (child-days)

Variable	Sprinkle (n=7,878 child-days)	Fortified complementary food (n=9,532 child-days)
Days fortified food not offered (n, %)	2,841 (36.1)	1,772 (18.6)
Days fortified food offered (n, %)	5,037 (63.9)	7,760 (81.4)
Mother's reasons for not offering food		
Did not want to give	416 (14.66)	260 (14.7)
Forgot to give	1,970 (69.44)	1,143 (64.9)
Child fell sick	451 (15.91)	369 (20.9)
No. of times fortified food offered	5,980 (76.3)	9,051 (96.8)
Amount of fortified food offered daily		
All	4,594 (91.9)	7,265 (95.7)
≥Half	380 (7.6)	307 (4.0)
<Half	23 (0.5)	16 (0.03)
Amount of fortified food eaten daily		
All	4,028 (80.6)	6,214 (81.9)
≥Half	667 (13.4)	986 (13.0)
<Half	152 (3.0)	302 (4.0)
None	150 (3.0)	86 (1.1)
Mother's perception of child's appetite		
Good	6,874 (87.7)	8,669 (92.3)

## DISCUSSION

In this study, we found that both strategies of food fortification in the form of rice-based fortified complementary food and sprinkle added to food were acceptable for sustained use for a period of 6 months. We recorded a long-term compliance of more than 60% among children in both the groups. Rice-based fortified complementary food was consumed for more than 80% of the child-days, which is considered a high compliance in intervention studies (29). Given the design of the study, the results reflect comparison of sprinkle or fortified complementary food with education compared to education alone and, therefore, evaluate additional impact of two interventions beyond education and not in comparison with education.

**Table 4.** Effect of food fortification on change in haematological markers and growth indices

Paired differences	Sprinkle (Mean±SD)	Control (Mean±SD)	Mean difference (95% CI)	p value	Fortified complementary food (Mean±SD)	Control (Mean±SD)	Mean difference (95% CI)	p value
Haematological markers	(n=62)	(n=48)			(n=66)	(n=48)		
Hb (g/dL)	0.37±1.1	0.23±1.3	0.15 (-0.29,0.59)	0.5	1.29±1.6	0.23±1.3	1.07 (0.51,1.62)	<0.001
HCT (%)	0.65±2.7	0.85±3.2	−0.20 (-1.3,0.9)	0.7	3.20±4.4	0.85±3.2	2.41 (0.91,3.90)	0.002
MCV (fl)	−0.008±8.8	1.57±6.8	−1.58 (-4.67,1.50)	0.3	4.30±8.3	1.57±6.8	2.71 (-0.21,5.64)	0.07
RDW (%)	−0.63±2.8	−0.98±2.7	0.34 (-0.71,1.4)	0.5	−1.25±2.5	−0.98±2.7	−0.26 (-1.24,0.71)	0.59
Growth indices	(n=49)	(n=53)			(n=59)	(n=53)		
WLZ	0.91±0.99	0.66±0.81	0.25 (-0.11,0.62)	0.17	0.79±0.86	0.66±0.81	0.13 (-189,0.453)	0.4
WAZ	0.65±0.79	0.52±0.67	0.12 (-0.17,0.40)	0.4	0.72±0.81	0.52±0.67	0.19 (-0.85,0.48)	0.17
LAZ	−0.22±0.46	−0.21±0.47	−0.003(-0.19,-0.18)	0.97	−0.05±0.49	−0.21±0.47	0.16 (-0.02,0.34)	0.07
Weight velocity (kg/yr)	1.45±0.53	1.39±0.35	0.06 (-0.12,0.24)	0.5	1.51±0.51	1.39±0.35	0.12 (-0.39,0.28)	0.14
Length velocity (cm/yr)	4.20±1.50	4.50±1.20	−0.30 (-0.82,0.25)	0.29	4.9±1.4	4.50±1.20	0.30 (-0.16,0.86)	0.17

Hb=Haemoglobin; HCT=Haematocrit; MCV=Mean corpuscular volume; RDW=Red cell distribution width; WLZ=Weight-for-length; WAZ=Weigh-for-age; LAZ=Length-for-age

Lower compliance observed in micronutrient powder (sprinkle) compared to fortified complementary food could have been attributed to either change in taste and/or colour of the food in which it was added or due to mother forgetting to give the sprinkle powder. One of the possible explanations for the difference between sprinkle and fortified complementary food-group could have been because the sprinkle could only be used in conjunction with a meal while fortified complementary food could be offered stand-alone.

The commonly-used vehicles for centralized iron and zinc fortification are rice, wheat, or salt, wherein fortificants are added in microquantities to improve the nutrient content of the child's diet (30). Although all these vehicles can be used as an effective strategy for delivering supplements, the choice is often limited by availability and cost of the centralized food and the quantity of food that the child is consuming and mother's effort in cooking the fortified food. In this study, we evaluated an approach that could replace the need for supplementation, using food-based or local fortification approaches, to deliver micronutrients shown to have benefit using supplementation approaches.

Our findings on compliance with fortification are in concurrence with results of the fortification studies in other developing countries. Recent trials on home-fortification of complementary foods with micronutrient supplements in the form of powder, crushable tablets, or fat-based spreads have shown a similar compliance of more than 85% of child-days (31-34). Other studies based on micronutrient powder have shown mixed data on compliance of as low as 7% to a high compliance of 85%. The lower compliance often has been attributed to child not liking the change in taste and/or colour of the food to which it was added (21), or humidity rendering the sprinkle non-useable (24).

The approaches of fortification used in the study did not result in food displacement, which supports the fact that this strategy can augment the existing food habits and practices of the population. Similar findings were reported in a developed-country setting, using complementary cereal foods (35). As an additional outcome, adequate compliance with consumption of rice-based fortified complementary food was further reflected in improved iron status as observed by improvement in iron status markers, like Hb, RDW, and MCV. Addition of a sprinkle had reduced effect-size on the haematological and anthropometric parameters. Studies on efficacy and bioavailability of fortification strategies using either micronutrient powder or fortified complementary food reported similar haematological improvements in developing-country settings where the prevalence of iron and zinc deficiency is high (18,20,21,36-39).

The present study suggests that micronutrient-fortified complementary food and the use of sprinkle could have sustained use and, thus, are suitable delivery mechanisms of iron and zinc in this population. Both the products are easy to deliver and mix; have high shelf-life; low-cost, do not alter the appearance, taste, and organoleptic properties of the food; has very less risk of overdose; and can easily provide the required quantities of iron and zinc to the child. Although addition of sprinkle slightly changed properties of food in terms of taste and colour on standing, the compliance would probably be better with micro-encapsulated iron. Adequate compliance with these home-based delivery strategies can be the key to effective delivery of iron and zinc in a population of high prevalence of micronutrient deficiency.

## ACKNOWLEDGEMENTS

The trial was funded by Thrasher Foundation and World Health Organization (WHO). We gratefully acknowledge the contributions of parents of children enrolled in the study and the study team, including health workers, supervisors, physicians, and data management and other support staff.
